# Fermented Fish Collagen Diminished Photoaging-Related Collagen Decrease by Attenuating AGE–RAGE Binding Activity

**DOI:** 10.3390/cimb46120860

**Published:** 2024-12-20

**Authors:** Seyeon Oh, So Young Lee, Jong-Won Jang, Kuk Hui Son, Kyunghee Byun

**Affiliations:** 1 Functional Cellular Networks Laboratory, Lee Gil Ya Cancer and Diabetes Institute, Gachon University, Incheon 21999, Republic of Korea; seyeon8965@gmail.com (S.O.); jh58333@gachon.ac.kr (J.-W.J.); 2 Department of Thoracic and Cardiovascular Surgery, Gachon University Gil Medical Center, Gachon University, Incheon 21565, Republic of Korea; faustina117@gilhospital.com; 3 Department of Health Sciences and Technology, Gachon Advanced Institute for Health & Sciences and Technology (GAIHST), Gachon University, Incheon 21999, Republic of Korea; 4 Department of Anatomy & Cell Biology, College of Medicine, Gachon University, Incheon 21936, Republic of Korea

**Keywords:** advanced glycation end products, AGE–RAGE binding activity, fermented fish collagen

## Abstract

Ultraviolet (UV) irradiation causes skin wrinkles and decreases elasticity. UV also increases binding between advanced glycation end products (AGEs) and the receptor for AGEs (RAGE), resulting in increased inflammation and activation of NF-κB. We evaluated whether fermented fish collagen (FC) could decrease photoaging via decreasing AGE–RAGE binding activity, which was associated with decreased TNF-α and NF-κB levels in UV-irradiated keratinocytes and animal skin. In the UV-irradiated keratinocytes, AGE–RAGE binding activity and TNF-α secretion levels were increased, and FC decreased these. Additionally, AGE–RAGE binding activity and TNF-α secretion levels were attenuated by soluble RAGE (RAGE inhibitor) in the UV-irradiated keratinocytes. FC decreased AGE–RAGE binding activity, TNF-α levels, and translocation of NF-κB in the UV-irradiated skin. Furthermore, FC decreased the expression of matrix metalloproteinases 1/3/9, which degrades collagen fibers, and Smad7, which inhibits Smad2/3, in UV-irradiated skin. FC increased Smad2/3 and collagen fiber accumulation. FC also increases skin moisture and elasticity. In conclusion, FC could attenuate skin photoaging via decreasing AGE–RAGE binding activity and its downstream signals such as TNF-α and NF-κB.

## 1. Introduction

Advanced glycation end products (AGEs) are generated by spontaneous reactions of proteins, nucleic acids, and lipids with glucose or reducing monosaccharides [1]. AGEs can accumulate in various tissues and the amount of accumulated AGE increases with age. Moreover, AGE accumulation increases in diabetes or chronic inflammation and is associated with the development of various diseases [2,3,4]. When cellular oxidative stress increases, reactive oxygen species (ROS) promote the oxidation of proteins, lipids, and nucleic acids, subsequently increasing AGE generation [5,6]. Chronic inflammation promotes glycation reactions that trigger AGE [7].

Ultraviolet (UV) irradiation leads to photoaging of the skin, which is the main extrinsic factor in skin aging, by increasing oxidative stress [7]. AGE accumulation is increased by UV irradiation in the epidermis and dermis [8]. UV light also promotes saccharification of fibers in the extracellular matrix (ECM), which triggers the accumulation of AGEs [5]. The receptor for AGEs (RAGE) is the principal receptor involved in AGE binding. Through binding of AGEs to RAGE, various signal pathways are activated that increase inflammation as well as propagation of ROS generation [3,9]. RAGE promotes activation of activator protein 1 (AP-1) and nuclear factor-kappa B (NF-κB), which upregulated tumor necrosis factor-alpha (TNF-α) and interleukin (IL)-6 [10,11].

UV irradiation also increased activation of NF-κB by increasing oxidative stress [12]. UV irradiation-induced NF-κB activation led to upregulation of matrix metalloproteinases (MMPs), which degrade ECM structures [13,14]. Transforming growth factor-β (TGF-β) is involved in collagen synthesis via phosphorylating transcription factors of Smad2 and Smad3 [15,16]. UV irradiation also led to decreased collagen synthesis by inhibiting TGF-β and Smad2/3 pathways [17]. Differently from Smad2/3, Smad7 inhibits TGF-β via preventing phosphorylation of Smad2/3 [18,19,20]. TNF-α activates NF-κB, which increases Smad7 [21]. Smad7 subsequently inhibits TGF-β in the fibroblast [21].

Fermented fish collagen (FC) is a low-molecular-weight collagen fermented using specialized Lactobacillus strains (*Lactobacillus plantarum* BJ21 and *Lactobacillus brevis* BJ20). It contains various peptides including glycine. Glycine serves as a precursor for glutathione (GSH), a primary endogenous antioxidant [22]. A previous study revealed that glycine administration decreases the AGE/RAGE signaling pathway in the aorta of diabetic rats [23].

Since decreasing oxidative stress led to decreased AGE formation, various antioxidants have been evaluated for decreasing AGE level. In fact, polyphenol compounds and various antioxidants, such as vitamin C, have also been shown to decrease skin photoaging [24]. AGE production could be inhibited by blocking each glycation step. Thus, AGE inhibitors can be classified into the following categories: (1) carbonyl trapping agents that reduce carbonyl stress, (2) metal ion chelators or free radical scavengers that inhibit sugar and lipid oxidation reactions, (3) crosslinking breakers that reverse AGE crosslinking, (4) agents that activate the endogenous antiglycation system, and (5) RAGE antagonists, such as soluble RAGE (sRAGE) [24]. sRAGE is formed by proteolytic cleavage of the extracellular domain of the RAGE cell surface receptor [25]. sRAGE can act as a decoy of RAGE, sequestering AGEs by binding to AGEs and subsequently inhibiting RAGE signaling [26]. Because AGE formation is a complex process, blocking only one step of glycation is insufficient to decrease AGE formation.

Although RAGE induces photoaging of the skin and glycine decreases RAGE activity, whether FC decreases RAGE pathway activity, which could promote photoaging, has not yet been revealed. Thus, we aimed to evaluate whether FC decreases photoaging by decreasing RAGE activity in a UV-irradiated animal model. We hypothesized that FC decreased RAGE, which decreased TNF-α and NF-κB, which subsequently decreased MMPs and Smad7. Decreased Smad7 activity leads to increased Smad2/3 expression, which, in turn, increases collagen synthesis. Furthermore, decreased MMP levels lead to decreased destruction of the ECM which, in turn, leads to decreased photoaging.

## 2. Materials and Methods

### 2.1. FC Preparation and Analysis

#### 2.1.1. FC Preparation

The FC sourced from GABALAGEN was procured from Marine Bioprocess Co., Ltd. (Busan, Republic of Korea). Prior to continuing with the fermentation process, fish collagen supplied and obtained from Geltech Co., Ltd. (Busan, Republic of Korea) underwent hydrolysis at a temperature of 55 ± 2 °C for a duration of 12 h, facilitated by a prozyme provided by Bisionbiochem Co., Ltd. (Seoul, Republic of Korea). The production of FC was achieved through two fermentations stages using *Lactobacillus brevis* BJ20 (accession No. KCTC 11377BP) and *Lactobacillus plantarum* BJ21 (accession No. KCTC 18911P).

The seed medium was prepared with 3% yeast extract and 1% glucose (both from Choheung, Republic of Korea), along with 1% L-glutamic acid (Samin Chemical, Siheung, Republic of Korea) and 95% water. This mixture was sterilized at 121 °C for 15 min prior to inoculation with 0.002% of each strain. The microorganisms were incubated separately at 37 °C for 24 h.

In the first fermentation stage, a culture medium containing 10% (*v/v*) *L*. *brevis* BJ20 was combined with a fermentation medium composed of 2% yeast extract, 0.28% glucose, 29% hydrolyzed fish collagen (Geltech Co., Ltd.), 5.5% L-glutamic acid (Samin Chemical, Siheung, Republic of Korea), and 63.22% water. The mixture was fermented at 37 °C for 24 h. Following this, 10% (*v/v*) of the pre-cultured seed medium of *L*. *plantarum* BJ21 was introduced, and fermentation proceeded at 37 °C for another 24 h. Finally, the fermentation medium was sterilized and subjected to spray drying to yield and produce FC powder samples.

#### 2.1.2. High-Performance Liquid Chromatography (HPLC) Evaluation

To prepare a standard solution, a standard sample weighing 0.1 g was dissolved in 100 mL distilled water (DW) in a volumetric flask. The solution was then filtered using a polytetrafluoroethylene syringe filter (25 mm/0.2 μm) and stored at −80 °C. A five percent aqueous sample solution was prepared by dissolving 5 g of FC in DW in a 100 mL volumetric flask and subsequently filtered by the same syringe filter.

Analysis was performed using a Dionex U3000 series HPLC system (Thermo Fisher Scientific, Waltham, MA, USA), equipped with a UV detector, operating at a flow rate of 1 mL/min. The samples were analyzed using UV–vis spectrophotometry at a wavelength of 338 nm (Figure S5). The concentrations of γ-aminobutyric acid (GABA) and glycine in the FC were calculated using the following formula:

Substance (mg/g) = Measurement (mg/mL) × Dilution factor ÷ Amount (g) × 100 (mL).

The FC produced consisted of 1% (*w/w*) glycine and 9% (*w/w*) GABA.

### 2.2. In Vitro Model

Human keratinocytes (HaCaT) were generously provided by Professor Jeong Hee Hong’s research group at Gachon University. The keratinocytes were grown in Dulbecco’s Modified Eagle Medium (HyClone, Logan, UT, USA) under controlled conditions of 37 °C with 5% CO_2_.

Several in vitro experiments were conducted to evaluate the effects of FC and glycine on keratinocytes. Initially, the cytotoxicity of FC was assessed by culturing keratinocytes until they reached 70% confluence. The cells were then washed with phosphate-buffered saline (PBS, Gibco, Thermo Fisher, Waltham, MA, USA) and treated with various concentrations of FC, ranging from 1 to 100,000 μg/mL, for the CCK-8 assay (Transgene Biotech Co., Beijing, China; Figure S1).

To identify the optimal concentration of FC, keratinocytes were subjected to UV irradiation (peak wavelength 306 nm) for 30 s, followed by treatment with PBS or FC at concentrations of 50, 100, 250, and 500 μg/mL for 48 h. The supernatant was then collected to analyze TNF-α secretion (Figure S2).

To establish the optimal concentration of sRAGE, keratinocytes were irradiated with UV light for 30 s and subsequently treated with PBS or sRAGE (Biovendor, Brno, Czech Republic) at concentrations of 50, 100, 200, and 400 ng/mL for 24 h. The supernatant was collected for TNF-α secretion analysis (see Figure S3).

After determining the optimal concentrations, keratinocytes were subjected to UV irradiation and treated with 50 ng/mL sRAGE for 24 h. This was followed by treatment with either PBS, 250 μg/mL of FC, or 2.5 μg/mL of glycine (Sigma-Aldrich, St. Louis, MO, USA) for a continued 48 h. Control groups not exposed to UV irradiation were incubated with PBS under identical conditions. Following the 48 h incubation, both cell lysates and supernatants were gathered for protein analysis (see Figure S6).

### 2.3. In Vivo Model

#### 2.3.1. Mouse Model and Maintenance

Six-week-old female hairless HRM-2 mice were acquired from the Central Laboratory Animal Center (Seoul, Republic of Korea) and acclimated in our animal center for two weeks prior to the start of the experiments. The mice were maintained under controlled conditions, with a stable temperature range of 20–24 °C and atmospheric moisture between 45 and 55% and had unrestricted access to food and water. The study protocol was approved by the Animal Experiment Ethics Committee at Gachon University (IACUC, approval code: LCDI-2022-0107).

#### 2.3.2. Experimental Design

After acclimatization, the animals were assigned to five groups at random. Four of these classifications were exposed to UV irradiation following previously described protocols [27]. In brief, the backs of the mice were irradiated with 200 mJ/cm^2^ of UV light from a UV lamp (peak wavelength 306 nm; Sankyo Korea Co., Ltd., Suwon, Republic of Korea) every two days for ten days, then daily irradiation for three consecutive days. Afterward, the mice were administered either water or FC orally at 150, 250, or 350 mg/kg (5 mL/kg of body weight) and exposed to UV light every other day for 28 days [28]. The FC was combined with drinking water, and its neutral taste and odor did not interfere with ingestion. Skin samples were collected from the animals at the end of the treatment period (see Figure S7).

#### 2.3.3. Skin Moisture and Elasticity

Skin moisture and elasticity were assessed using an API-100 device (Aram HUVIS, Seongnam, Republic of Korea). Measurements were taken five times on the 42nd day after the initial UV exposure, which was also the 28th day following the start of treatment. The average values from these measurements were then calculated.

### 2.4. Sample Preparation

#### 2.4.1. Protein Isolation

Per the manufacturer’s protocol, proteins were extracted using an EzRIPA Lysis Kit (ATTO Corp., Tokyo, Japan). The cells were rinsed with PBS and resuspended in 1 mL of RIPA buffer. For skin samples, 50 mg of tissue was finely minced, mixed in 0.6 mL of RIPA buffer, and homogenized through sonication for ten cycles (40 s on, and 60 s off). This mixture was incubated on ice for 10 min to improve protein solubility. Both cell and tissue samples were subsequently sonicated again (high power, 10 s on, 60 s off) and centrifuged at 14,000× *g* for 15 min at four degrees to extract the proteins. Protein concentrations were assessed using a bicinchoninic acid assay kit from Thermo Fisher Scientific.

#### 2.4.2. Paraffin-Embedded Skin Tissue Specimen

Skin tissues were preserved in cold 4% paraformaldehyde (Sigma-Aldrich) for a duration of 48 h, then transferred to cassettes, rinsed with DW, and processed through a series of dehydration procedures utilizing a tissue processor (Leica, Wetzlar, Germany). The tissues were progressively soaked in 95% and 99% ethanol (Duksan, Ansan, Republic of Korea) and xylene (Duksan), before being embedded in paraffin (Leica). The paraffin-embedded tissue samples were subsequently shaped into blocks with an embedding machine, sliced to a thickness of 7 μm using a microtome (Leica), placed on pre-coated slides, incubated overnight at 60 °C, and allowed to adhere to the slides.

### 2.5. Cell Viability

To evaluate the cytotoxicity of FCs, keratinocytes were cultured in 96-well plates at a density of 10,000 cells per well. After reaching full confluence, the cells were exposed to FCs at concentrations ranging from 1 to 100,000 μg/mL for 24 h. The medium was removed after treatment, and cells were gently washed with Dulbecco’s PBS (Gibco). Subsequently, 10 μL of CCK-8 reagent (Transgene Biotech Co.) along with 90 μL of growth medium was added to each well. The plates were incubated at 37 °C for 2 h. The optical density was measured at 450 nm using a microplate reader (Thermo Fisher Scientific). All experiments were conducted in triplicate to ensure consistent and reliable results.

### 2.6. Enzyme-Linked Immunosorbent Assay

Microplates were initially incubated overnight at four degrees with 100 nM carbonate and bicarbonate mixed buffer (pH 9.6; Sigma-Aldrich) and then washed three times with PBS containing 0.1% Tween 20 (LPS solution, Daejeon, Republic of Korea) (TPBS) to eliminate unbound substances. To prevent nonspecific protein binding, the plates were blocked overnight at 4 °C with 5% skim milk (LPS solution) dissolved in 0.1% TPBS. After washing three more times with 0.1% TPBS, each well received 30 μg of either the cell lysate or tissue protein sample and was incubated overnight at four degrees. Following another wash with 0.1% TPBS, primary antibodies diluted in PBS (see Table S1 for details) were added to the wells and incubated overnight at four degrees. After a final PBS wash, horseradish peroxidase-conjugated secondary antibodies (1:1000; Vector Laboratories, Burlingame, CA, USA) were introduced, and the plates were kept in the dark at room temperature for four hours. Protein expression was visualized by adding tetramethylbenzidine solution (Sigma-Aldrich) to each well and incubating at room temperature in the dark for 15–20 min. The reaction was terminated by adding sulfuric acid (Sigma-Aldrich), and absorbance was measured at 450 nm with a microplate reader. Absorbance values were normalized against a Control group, and each experiment was conducted in triplicate for reliability and precision.

### 2.7. Western Blot

Thirty micrograms of either cell lysate or skin protein were combined with 4× LDS sample buffer and 10× sample reducing agent (both from Thermo Fisher Scientific). The mixture was heated at 70 °C for 10 min to denature the proteins. Protein was then separated using a 10% sodium dodecyl sulfate-polyacrylamide gel electrophoresis (SDS-PAGE) gel at 200 V for 25 min in MOPS buffer (Invitrogen, Waltham, MA, USA). After separation, proteins were transferred to PVDF membranes (Millipore, Burlington, MA, USA) using a semi-dry transfer system (ATTO) at 1 A for 10 min. To prevent nonspecific binding, the membranes were blocked with Tris-buffered saline (TTBS) containing 0.1% Tween 20 (LPS solution) and 5% skim milk (LPS Solution) for 1 h at room temperature. Following three washes with 0.1% TTBS, membranes were incubated overnight at 4 °C with primary antibodies (as listed in Table S1). After three additional washes with 0.1% TTBS, membranes were incubated for 1 h at room temperature with horseradish peroxidase-conjugated secondary antibodies (1:10,000; Vector Laboratories).

Protein bands were detected using a chemiluminescent solution and visualized on a ChemiDoc Imaging System (Bio-Rad, Hercules, CA, USA). Band intensity was quantified using ImageJ software version 1.53s (NIH, Staten Island, NY, USA), using beta-actin as a loading control to ensure equal samples loading. Each experimental group was compared to the control group to evaluate changes in protein expression.

### 2.8. Staining

#### 2.8.1. 3,3′-Diaminobenzidine Staining (DAB)

For antigen retrieval, the slides were heated in a microwave oven for 5 min in sodium citrate buffer (pH 6.0; Sigma-Aldrich), cooled in DW and washed with PBS. The slides were treated with 3% hydrogen peroxide in PBS for 10 min at room temperature, followed by three PBS washes. To block endogenous peroxidase activity, slides were incubated with 0.1% Triton X-100 (Sigma-Aldrich) in PBS for 5 min at room temperature, washed with PBS, and then incubated with 0.01% normal serum solution (Vector Laboratories) for 1 h to prevent nonspecific binding. The blocked slides were incubated overnight at four degrees with primary antibodies (refer to Table S1), followed by a 1 h incubation at room temperature. After washing, biotinylated secondary antibodies (Vector Laboratories) were applied for 1 h at room temperature, washed with PBS, and treated with the ABC reagent (Vector Laboratories) according to the manufacturer’s protocol. After another PBS wash, slides were developed using 3,3′-diaminobenzidine solution (Sigma-Aldrich) for 15 min to produce a brown color. For counterstaining, the slides were immersed in hematoxylin (KPNT; Cheongju, Republic of Korea) for 30 s, washed with DW, and mounted using DPX mounting solution (Sigma-Aldrich). The slides were then scanned using a slide scanner (Motic Scan Infinity 100, Motic, Beijing, China), and the intensity and positive signals were quantified using ImageJ software version 1.53s [29,30].

#### 2.8.2. Masson Trichrome Staining

To stain collagen fibers in skin tissue, sections that were previously fixed and embedded in paraffin were immersed in Bouin’s solution (Scytek Laboratories, West Logan, UT, USA), heated at 60 °C for 1 h, followed by a rinse with DW. The sections were then treated sequentially at room temperature with ferrous hematoxylin (Scytek Laboratories) for 10 min, Biebrich–Scarlet acid fuchsin solution (Scytek Laboratories, West Logan, UT, USA) for 2 min, phosphomolybdenum-phosphotungstic acid solution (Scytek Laboratories) for 15 min, and aniline blue solution (Scytek Laboratories) for 3 min. After staining, the slides were sealed with DPX mounting solution (Sigma-Aldrich) and examined under a light microscope (Olympus, Tokyo, Japan) equipped with a slide scanner (Motic). The density of collagen fiber in the images was analyzed using images using ImageJ software version 1.53s [31].

#### 2.8.3. Herovici Staining

A Herovici Collagen Stain Kit (Scytek Laboratories) was employed to distinguish between mature and newly formed collagen fibers for analyzing skin tissue samples. Deparaffinized slides were first incubated in Weigert iron hematoxylin for 8 min to stain the nuclei, then rinsed with tap water and DW. The slides were treated with Herovici solution for 2 min before being sealed with DPX mounting solution (Sigma-Aldrich). Microscopy and imaging were conducted using a slide scanner (Motic). In this staining method, newly synthesized collagen fibers appear blue, while mature collagen fibers appear red [32,33]. Quantitative analysis of the staining was carried out using ImageJ software version 1.5.

### 2.9. Statistical Analysis

Group comparisons were conducted using Kruskal–Wallis tests, with subsequent Mann–Whitney U tests utilized for post-hoc analysis. Results are presented as mean ± SD. All statistical analyses were carried out using SPSS version 26 (IBM Corp., Armonk, NY, USA), and statistical significance is noted in the figure legends.

## 3. Results

### 3.1. FC Decreased RAGE Expression and TNF-α in the UV-Irradiated Human Keratinocyte

**Figure 1:**
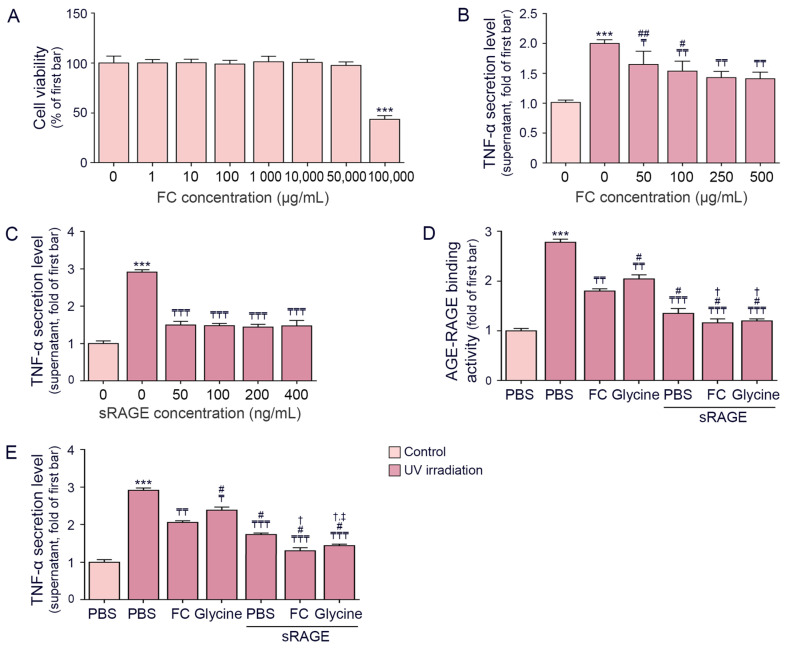
Downregulation of AGE–RAGE binding activity and TNF-α secretion by FC and glycine in UV-exposed keratinocytes. (**A**) Assessment of keratinocyte viability following treatment with FC. (**B**) Evaluation of TNF-α secretion in the supernatant of UV-exposed keratinocytes to determine the optimal concentration of FC. (**C**) Evaluation of TNF-α secretion in the supernatant of UV-exposed keratinocytes to determine the optimal concentration of sRAGE. (**D**) Analysis of AGE–RAGE binding activity in UV-exposed keratinocytes treated with sRAGE, FC, and glycine. (**E**) Measurement of TNF-α secretion level in the supernatant of UV-exposed keratinocytes following treatment with sRAGE, FC, and glycine. Data are expressed as mean ± SD from three independent experiments. ***, *p* < 0.001, vs. Control/PBS; ₸, *p* < 0.05, ₸₸, *p* < 0.01, ₸₸₸, *p* < 0.001, vs. UV/PBS; #, *p* < 0.05, ##, *p* < 0.01 vs. UV/FC (250 μg/mL); †, *p* < 0.05 vs. UV/sRAGE/PBS; ‡, *p* < 0.05 vs. UV/sRAGE/FC (Mann–Whitney U test). AGE, advanced glycation end products; FC, fermented fish collagen; PBS, phosphate-buffered saline; SD, standard deviation; RAGE, receptor for AGEs; sRAGE, soluble RAGE; TNF-α, tumor necrosis factor-alpha; UV, ultraviolet.

The optimal concentration of FC for treatment was determined by evaluating cell viability and its effectiveness in decreasing TNF-α levels. No significant decrease in cell viability of FC-treated keratinocytes was observed up to a concentration of 50,000 μg/mL, while cell death was observed at concentrations of 100,000 μg/mL (Figure 1A and Figure S1).

UV irradiation resulted in an increase in TNF-α secretion level, which was decreased by administration of FC. We observed that TNF-α reduction between 250 and 500 μg/mL was not significantly different (Figure 1B and Figure S2). Therefore, we used 250 μg/mL FC for further experiments.

We then compared the effects of FC on RAGE activity and TNF-α secretion with those of glycine alone. Our findings revealed that the TNF-α-decreasing effect of FC was associated with decreased AGE–RAGE binding activity.

To determine the effective concentration of sRAGE that could decrease TNF-α secretion after UV irradiation, we treated sRAGE from 50 to 400 ng/mL with UV-irradiated keratinocytes. TNF-α secretion was significantly decreased from 50 ng/mL; thus, this concentration was determined as the treatment dose (Figure 1C and Figure S3).

The amount of AGE that bound to RAGE in keratinocytes was evaluated using a sandwich enzyme-linked immunosorbent assay. The binding activity of AGE to RAGE was increased by UV irradiation and decreased by FC and glycine. The effect of FC was greater than that of glycine. Treatment with sRAGE also decreased AGE–RAGE binding activity. Binding activity decreased further when sRAGE was treated with glycine or FC. The effect of FC on AGE–RAGE binding activity was weaker than that of sRAGE alone or sRAGE combined with FC or glycine (Figure 1D).

Secretion of TNF-α was increased by UV irradiation and decreased by FC, glycine, and sRAGE. The decreasing effects on TNF-α were highest when FC was treated with sRAGE (Figure 1E). These results suggested that FC or glycine decreased TNF-α in UV-irradiated keratinocytes and that the decrease in AGE–RAGE binding activity was involved in the TNF-α-decreasing mechanism.

### 3.2. FC Decreased RAGE, TNF-α, and NF-κB in UV-Irradiated Animal Skin

Since the decreasing effect of glycine on TNF-α was weaker than FC in the in vitro test, a comparison of the effect of FC with glycine was not performed in the animal study. Instead of comparing with glycine, FC concentration, which maximizes decreasing photoaging, was evaluated in the animal study.

**Figure 2:**
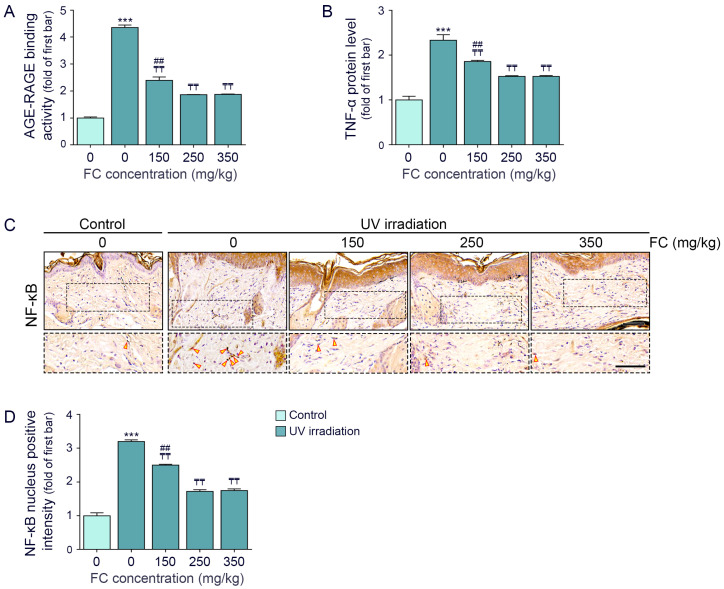
Downregulation of AGE–RAGE binding activity and TNF-α by FC in UV-exposed hairless mice skin. (**A**) Analysis of AGE–RAGE binding activity in the skin of UV-exposed hairless mice following FC treatments. (**B**) Assessment of TNF-α expression in the skin of UV-exposed hairless mice treated with FC treatments. (**C**,**D**) Examination of NF-κB translocation in the skin of UV-exposed hairless mice with FC treatments (The dashed box indicates an enlarged images and yellow arrow indicates positive signal; scale bar = 60 μm). Data are presented as the mean ± SD from three independent experiments. ***, *p* < 0.001, Control/water vs. UV/water; ₸₸, *p* < 0.01, vs. UV/water; ##, *p* < 0.01 vs. UV/FC 250 mg/kg (Mann–Whitney U test). AGE, advanced glycation end products; FC, fermented fish collagen; NF-κB, nuclear factor-kappa B; SD, standard deviation; RAGE, receptor for AGEs; TNF-α, tumor necrosis factor-alpha; UV, ultraviolet.

The amount of AGE that bound to RAGE increased with UV irradiation and decreased with FC treatment. No significant decrease in the amount of AGE that bound to RAGE was observed between the FC treatment groups of 250 and 350 mg/kg (Figure 2A).

TNF-α was increased by UV irradiation and it was decreased by FC. The decreasing effect between 250 and 350 mg/kg of FC was not significantly different (Figure 2B).

NF-κB activity was evaluated by the translocation of NF-κB into the nucleus. The expression of NF-κB in the nuclei was increased by UV irradiation and decreased by FC treatment. The effects of 250 and 350 mg/kg FC on NF-κB nuclear translocation were not significantly different (Figure 2C,D).

### 3.3. FC Decreased Expression of MMP1/3/9 and Smad7 in UV-Irradiated Animal Skin

**Figure 3:**
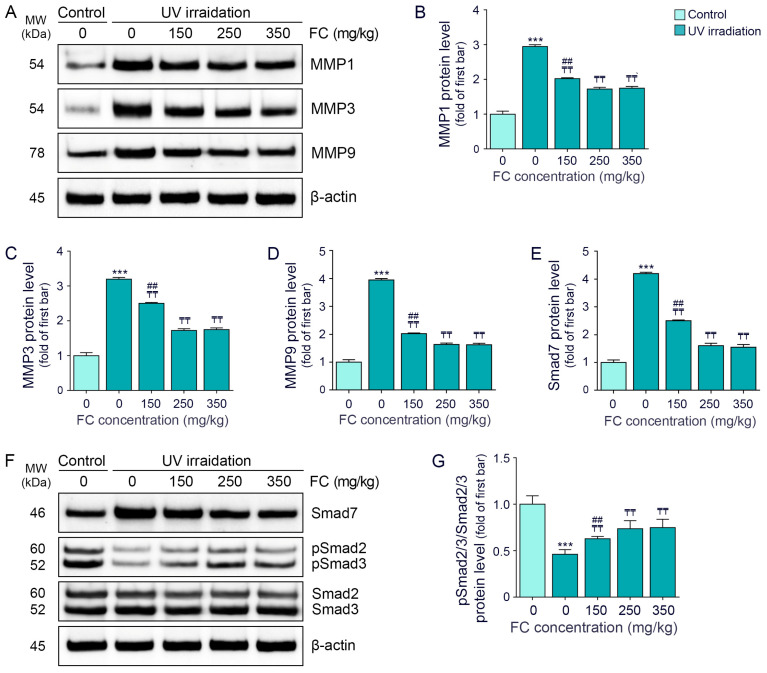
Modulation of MMPs and Smad expression by FC in UV-exposed hairless mice skin. (**A**) Analysis of MMPs protein expression in the skin of UV-exposed hairless mice following FC. (**B**–**D**) Quantitative evaluation of the Western blot data presented in panel (**A**). (**E**) Quantitative analysis of Smad7 Western blot results shown in panel (**F**). (**F**) Assessment of protein expression of Smad7, Smad2/3 and pSmad/3 in the skin of UV-exposed hairless mice treated with FC. (**G**) Quantitative evaluation of pSmad/3/Smad2/3 Western blot data presented in panel (**F**). Data are shown as the mean ± SD from three independent experiments. ***, *p* < 0.001, Control/water vs. UV/water; ₸₸, *p* < 0.01, vs. UV/water; ##, *p* < 0.01 vs. UV/FC 250 mg/kg (Mann–Whitney U test). β-actin, beta-actin; FC, fermented fish collagen; kDa, kilodalton; MMP, matrix metalloproteinase; MW, molecular weight; SD, standard deviation; Smad, suppressor of mothers against decapentaplegic; UV, ultraviolet.

UV irradiation increased the levels of MMP1/3/9, which were subsequently decreased by FC treatment. The decreasing effect of 250 and 350 mg/kg FC was not significantly different (Figure 3A–D).

The expression of Smad7 was increased by UV irradiation, which decreased by FC treatment. Similar to MMP levels, the reduction in Smad7 expression was not significantly different between the 250 and 350 mg/kg FC treatment groups (Figure 3E,F).

The ratio of phosphorylated Smad2/3 to total Smad2/3 was decreased by UV irradiation and increased following FC treatment. There was no significant difference in the ratio between the 250 and 350 mg/kg FC groups (Figure 3F,G).

### 3.4. FC Increased Collagen Accumulation in the UV-Irradiated Skin

**Figure 4:**
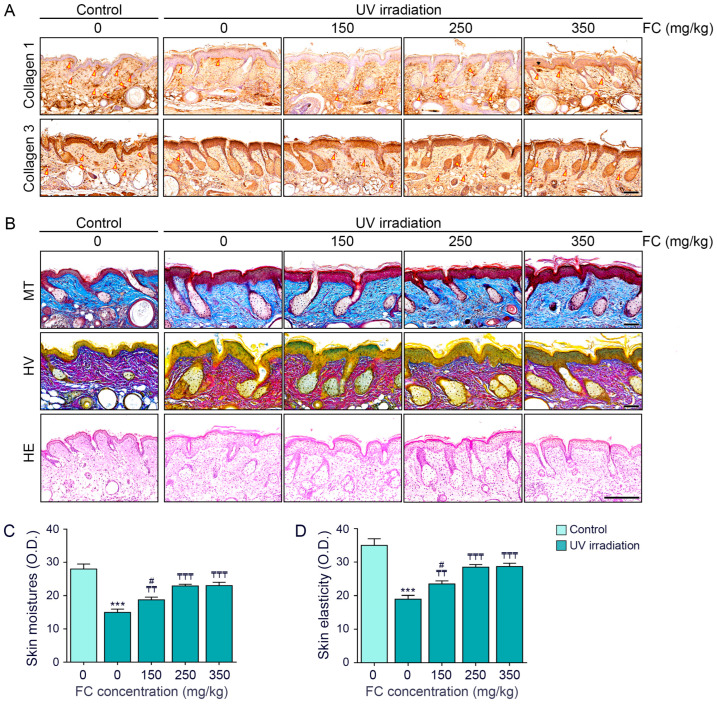
Upregulation of collagen accumulation by FC in UV-exposed hairless mice skin. (**A**) Immunohistochemical analysis of collagen type 1 and 3 in the skin of UV-exposed hairless mice treated with FC (yellow arrow indicates positive signal; scale bar = 100 μm). (**B**) Staining results from Masson trichrome, Herovici, and hematoxylin and eosin staining in the skin of UV-exposed hairless mice treated with FC (scale bar of MT and HV = 60 μm; scale bar of HE = 200 μm). (**C**,**D**) Quantitative evaluation of skin moisture and elasticity. Data are expressed as mean ± SD from three independent experiments. ***, *p* < 0.001, Control/water vs. UV/water; ₸₸ and ₸₸₸, *p* < 0.01 and *p* < 0.001, vs. UV/water; #, *p* < 0.05 vs. UV/FC 250 mg/kg (Mann–Whitney U test). FC, fermented fish collagen; HE, hematoxylin and eosin; HV, Herovici; MT, Masson trichrome; O.D., optical density; SD, standard deviation; UV, ultraviolet.

Collagen 1 and 3 levels were decreased by UV irradiation and increased by FC treatment. There was no significant difference in the effects of FC at 250 and 350 mg/kg (Figure 4A and Figure S4A,B).

Collagen fiber accumulation was evaluated using Masson trichrome staining, while Herovici staining was performed to stain the newly synthesized collagen (stained blue) [32,33]. Both collagen fiber accumulation and newly synthesized collagen levels were decreased by UV irradiation and increased by FC treatment. The effects of FC at 250 and 350 mg/kg of FC were not significantly different (Figure 4B and Figure S4C,D).

The thickness of the epidermis decreased with UV irradiation and increased with FC treatment. The effects of FC at 250 and 350 mg/kg of FC were not significantly different (Figure 4B and Figure S4E).

Skin moisture and elasticity were evaluated using the API-100 device. Skin moisture and elasticity decreased with UV irradiation and increased with the FC treatment. There was no significant difference between the effects of FC at 250 and 350 mg/kg on skin moisture and elasticity (Figure 4C,D).

## 4. Discussion

Skin photoaging can be clinically classified into two categories: hypertrophic and atrophic types [34]. Critical features of the hypertrophic type include skin laxity, deep and coarse wrinkling, and solar elastosis [34]. The atrophic type shows smooth, shiny skin accompanied by redness and telangiectasia [34]. The molecular mechanisms underlying photoaging are associated with UV-induced oxidative stress [35]. UV irradiation increases ROS, which upregulates MAPK and its downstream pathways of increased inflammatory cytokines such as IL-1, TNF-α, and IL-6 [36,37]. UV irradiation also promotes MMPs via the upregulation of MAPK, AP-1, and NF-κB [38,39,40]. Glycation of skin proteins is also involved in skin photoaging [41]. A glycated dermal matrix increases tissue stiffness and decreases skin elasticity [42].

Moreover, AGE–RAGE interactions in the epidermis lead to increased production of pro-inflammatory cytokines and ROS in keratinocytes [43]. AGE leads to apoptosis of fibroblasts, which are the main cells that produce ECM materials [44].

Since increased oxidative stress and inflammation lead to photoaging, several studies have been conducted to reduce photoaging by decreasing oxidative stress [24].

Collagen supplements are frequently administered orally for skin rejuvenation [45]. After ingestion, collagen supplements are hydrolyzed to dipeptides, tripeptides, or free amino acids, such as hydroxyproline, proline, and glycine [46,47]. Because these peptides are the main components of collagen synthesis, they can be used for collagen synthesis in the skin after absorption [45].

FC is a fermented collagen that contains glycine. Glycine is the most abundant amino acid in collagen and is necessary for maintaining skin elasticity [48]. Glycine is a fundamental antioxidant that inhibits the AGE/RAGE pathway by decreasing oxidative stress [23].

As FC contains glycine and collagen, we expected that FC would decrease photoaging by decreasing collagen destruction via the AGE–RAGE pathway. The UV-irradiated in vitro model showed that UV increased AGE–RAGE binding activity and TNF-α. Both were decreased by FC treatment. The decreasing effect of FC on AGE–RAGE binding activity and TNF-α was higher than that of glycine and lower than that of sRAGE. These results suggest that FC decreased the UV irradiation-induced TNF-α increase and that the AGE–RAGE pathway was involved in the decreasing process. Since oxidative stress increases the formation of AGE, FC might decrease AGE formation via decreasing oxidative stress. Moreover, RAGE expression was also increased by oxidative stress [49]. Activity of the glyoxalase system, which inhibits formation of AGE, was also decreased by oxidative stress [50]. Since glycine is a well-known antioxidant, it can be assumed that FC which contain glycine decreased AGE–RAGE binding activity by decreasing oxidative stress. We only evaluated the AGE–RAGE binding activity level in this study, thus the exact mechanism of FC on decreasing AGF-RAGE binding activity should be evaluated in the future study.

FC decreased AGE–RAGE binding activity more than glycine in our study. FC also contains hydroxyproline and glutamate [51], which are also reported to decreased oxidative stress [52,53]. Thus, the synergic effect of those factors increased the decreasing effect of FC on AGE–RAGE binding activity than single treatment of glycine. Similar to the in vitro study, UV-irradiated animal skin showed increased AGE–RAGE binding activity and TNF-α.

NF-κB is an inducible dimeric transcription factor that contains two major polypeptides, p65 and p50 [54]. NF-κB exists in the cytoplasm in an inactive form by binding with IκB, which is an inhibitory factor of NF-κB. UV or TNF-α leads to phosphorylation of IκB, resulting in the release of NF-κB from IκB [54]. NF-κB then translocates into the nucleus, leading to gene transcription [54]. UV-induced NF-κB activates various MMPs [55]. MMP1, MMP3, and MMP9 are the primary factors involved in epidermal photoaging [56].

MMP1 can almost completely degrade type I and III collagen, and MMP9 further degrades the collagen fiber fragments produced by MMP-1 [57]. MMP3 degrades type IV collagen, proteoglycans, and fibronectin [57].

In our study, UV treatment increased MMP1/3/9 levels, which were decreased by FC treatment.

Our results showed that NF-κB and Smad7 levels were increased by UV irradiation and decreased by FC treatment. Moreover, Smad2/3 phosphorylation was decreased by UV irradiation and increased by FC treatment. FC increased the accumulation of collagen fibers and newly synthesized collagen. The epidermal thickness was also decreased by FC. FC increases skin elasticity. Those effects were higher when FC of 250 mg/kg was administered than 150 mg/kg, not significantly different from 350 mg/kg.

Photoaging results in various skin changes, such as wrinkle formation, rough texture, decreased skin elasticity, and epidermal thickening [58,59]. Collagen supplements attenuate UV-induced decreases in skin hydration and increase epidermal thickening in animals [60]. Collagen supplements increase skin moisture and elasticity and decrease skin roughness [61]. In fibroblasts, collagen administration decreases MMP levels [62]. Similar to previous studies, our results showed that FC increased skin elasticity and moisture content. Although many trials have evaluated the effects of collagen supplements on skin rejuvenation, adverse effects have rarely been reported [63].

As FC was not administered for a long period of time, its long-term safety should be evaluated in future studies. As collagen was relatively safe in previous studies, FCs with similar components are expected to be safe.

## 5. Conclusions

In conclusion, FC reduced AGE–RAGE binding activity, TNF-α, and NF-κB, which was associated with decreased expression of MMP1, MMP3, and MMP8. Additionally, FC reduced Smad7 expression while increasing Smad2/3 expression, leading to enhanced collagen accumulation in UV-irradiated skin. FC effectively mitigated photoaging-related changes, such as reduced collagen accumulation and elasticity. While further mechanistic studies are needed to elucidate how FC reduces AGE–RAGE binding activity, this study demonstrated that FC prevents UV-induced collagen degradation in the skin. Given that FC is derived from fermented fish collagen and is expected to pose minimal risk upon consumption, its potential to enhance skin collagen production could offer valuable benefits from a skincare perspective.

## Data Availability

All data are contained within this article.

## Supplementary Materials

The following supporting information can be downloaded at: https://www.mdpi.com/article/10.3390/cimb46120860/s1, Figure S1: Schematic diagram showing the treatment of keratinocytes with FC for cytotoxic effects; Figure S2: Schematic diagram showing the treatment of UV-exposed keratinocytes with FC for suitable concentration; Figure S3: Schematic diagram showing the treatment of UV-exposed keratinocytes with sRAGE for suitable concentration; Figure S4: Regulation of collagen accumulation by FC in UV-exposed hairless mice skin; Figure S5: High-performance liquid chromatography analysis of GABA and Glycine in FC; Figure S6: Schematic diagram showing sRAGE treatment followed by FC or glycine treatment of UV-exposed keratinocytes; Figure S7: Schematic diagram showing the treatment of UV-exposed hairless mice skin with FC; Table S1: List of antibodies for enzyme-linked immunosorbent assay (ELISA), Western blot (WB) and 3,3′-diaminobenzidine staining (DAB).
