# Supplementary material for: Fermented Fish Collagen Diminished Photoaging-Related Collagen Decrease by Attenuating AGE–RAGE Binding Activity
Source: Curr Issues Mol Biol. 2024 Dec 20;46(12):14351–65. doi: 10.3390/cimb46120860 (PMC11674791; doi:10.3390/cimb46120860)
Supplement: Supplementary file 1 [file cimb-46-00860-s001.zip › cimb-3376624-supplementary.pdf]

current issues in molecular biology

MDPI

Article

Fermented fish collagen attenuated photoaging related collagen decrease by attenuating AGE-RAGE binding activity

Seyeon Oh [1,†], So Young Lee [2,†], Jong-Won Jang [1,3], Kuk Hui Son [2,*] and Kyunghee Byun [1,3,4,*]

[1] Functional Cellular Networks Laboratory, Lee Gil Ya Cancer and Diabetes Institute, Gachon University, Incheon 21999, Republic of Korea; seyeon8965@gmail.com
[2] Department of Thoracic and Cardiovascular Surgery, Gachon University Gil Medical Center, Gachon University, Incheon 21565, Republic of Korea; faustina117@gilhospital.com
[3] Department of Health Sciences and Technology, Gachon Advanced Institute for Health & Sciences and Technology (GAIHST), Gachon University, Incheon 21999, Republic of Korea; jh58333@gachon.ac.kr
[4] Department of Anatomy & Cell Biology, College of Medicine, Gachon University, Incheon 21936, Republic of Korea
[*] Correspondence: dr632@gachon.ac.kr (K.H.S.); khbyun1@gachon.ac.kr (K.B.); Tel.: +82-32-460-3666 (K.H.S.); +82-32-899-6511 (K.B.)
[†] These authors contributed equally to this work.

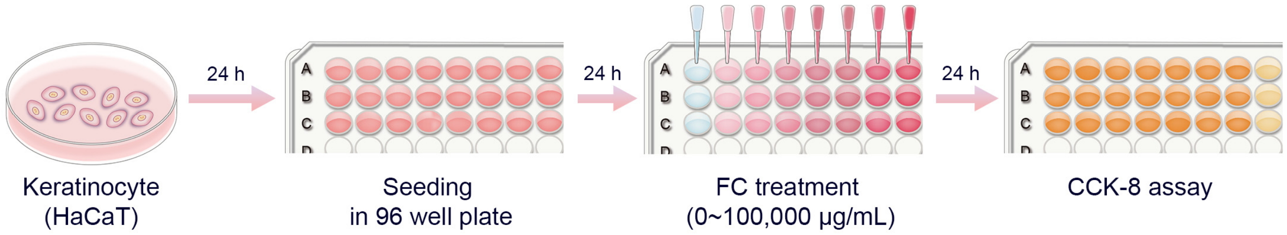

Figure S1. Schematic diagram showing the treatment of keratinocytes with FC for cytotoxic effects. CCK-8, cell counting kit-8; FC, fermented fish collagen; h, hours.

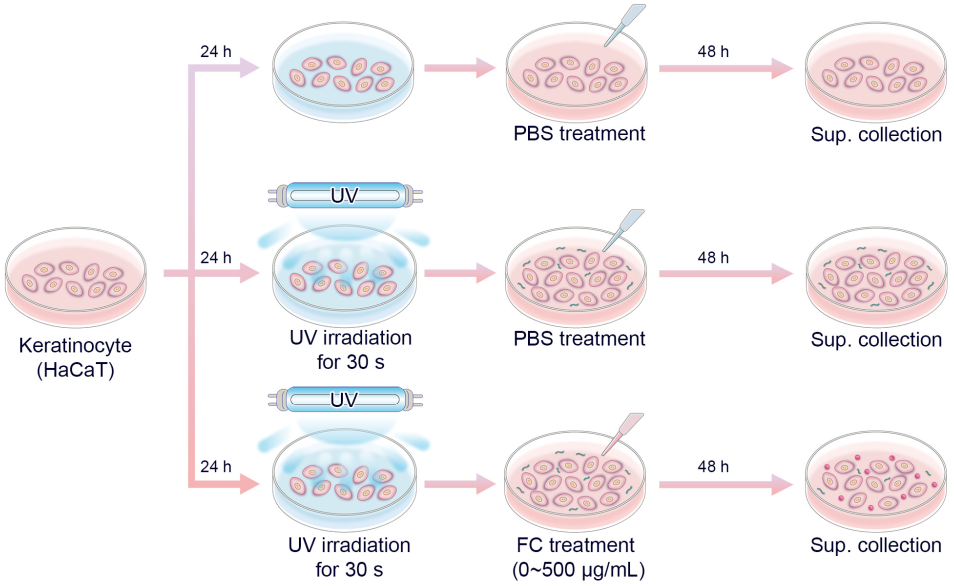

Figure S2. Schematic diagram showing the treatment of UV-exposed keratinocytes with FC for suitable concentration. FC, fermented fish collagen; h, hours; PBS, phosphate-buffered saline; s, seconds; Sup., supernatant; UV, ultraviolet.

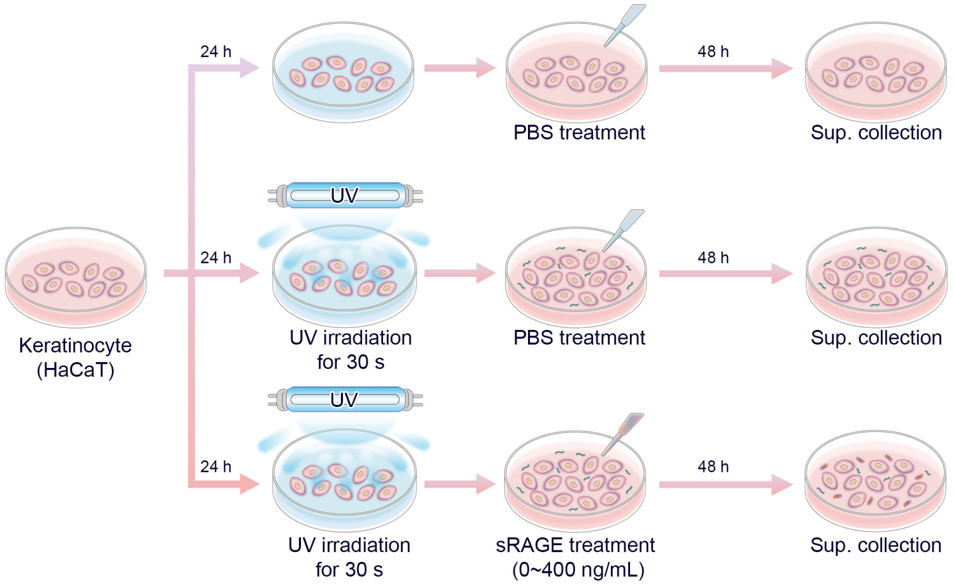

Figure S3. Schematic diagram showing the treatment of UV-exposed keratinocytes with sRAGE for suitable concentration. h, hours; PBS, phosphate-buffered saline; s, seconds; sRAGE, soluble receptor for advanced glycation end product; Sup., supernatant; UV, ultraviolet.

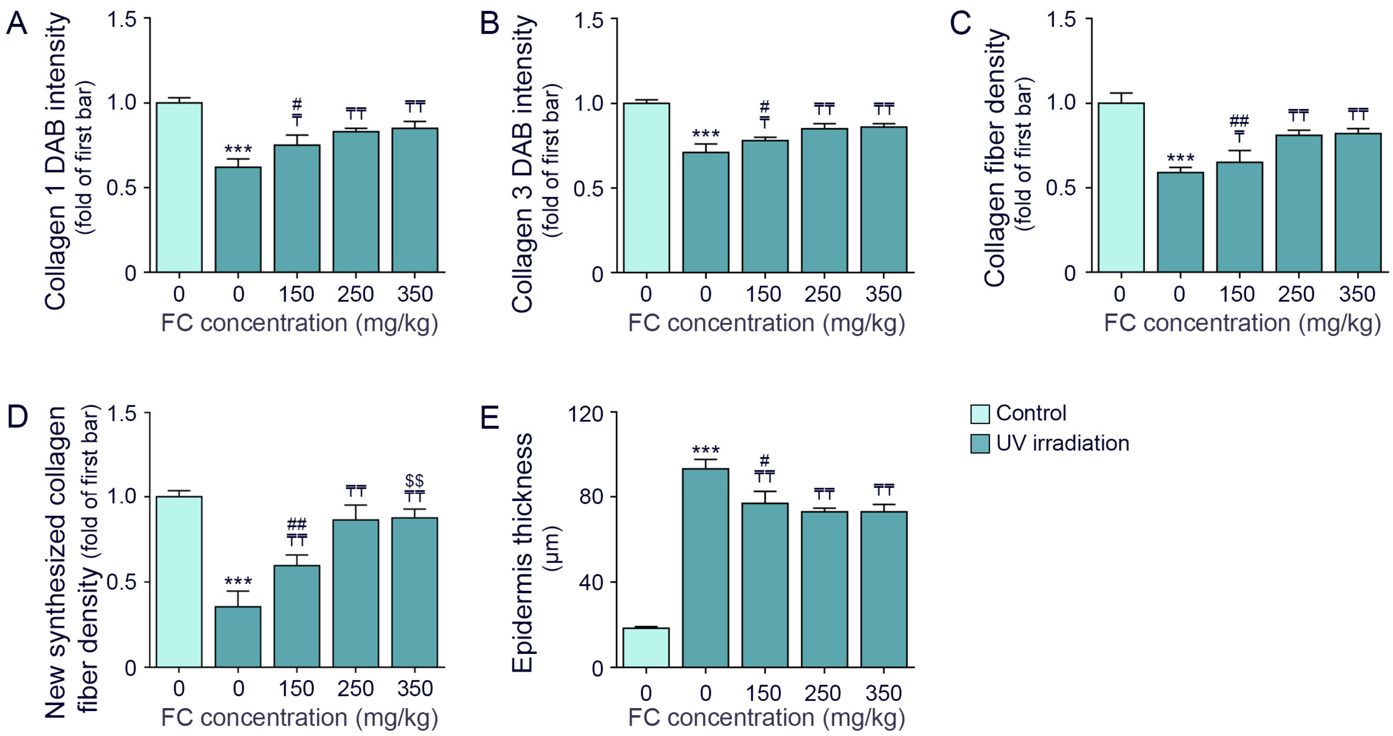

Figure S4. Regulation of collagen accumulation by FC in UV-exposed hairless mice skin. (**A**,**B**) Quantitative assessment of immunohistochemistry data presented in Figure 4A. (**C**) Quantitative assessment of masson trichrome stain data presented in Figure 4B upper line. (**D**) Quantitative assessment of herovici stain data presented in Figure 4B middle line. (**E**) Quantitative assessment of hematoxylin and eosin stain data presented in Figure 4B lower line. Data are presented as the mean ± SD of three independent experiments. ***, $p < 0.001$, Control/water vs. UV/water; ⊤ and ⊤⊤, $p < 0.05$ and $p < 0.01$, vs. UV/water; # and ##, $p < 0.05$ and $p < 0.01$ vs. UV/FC 250 mg/kg (Mann–Whitney U test). DAB, 3, 3'-diaminobenzidine; FC, fermented fish collagen; SD, standard deviation; UV, ultraviolet.

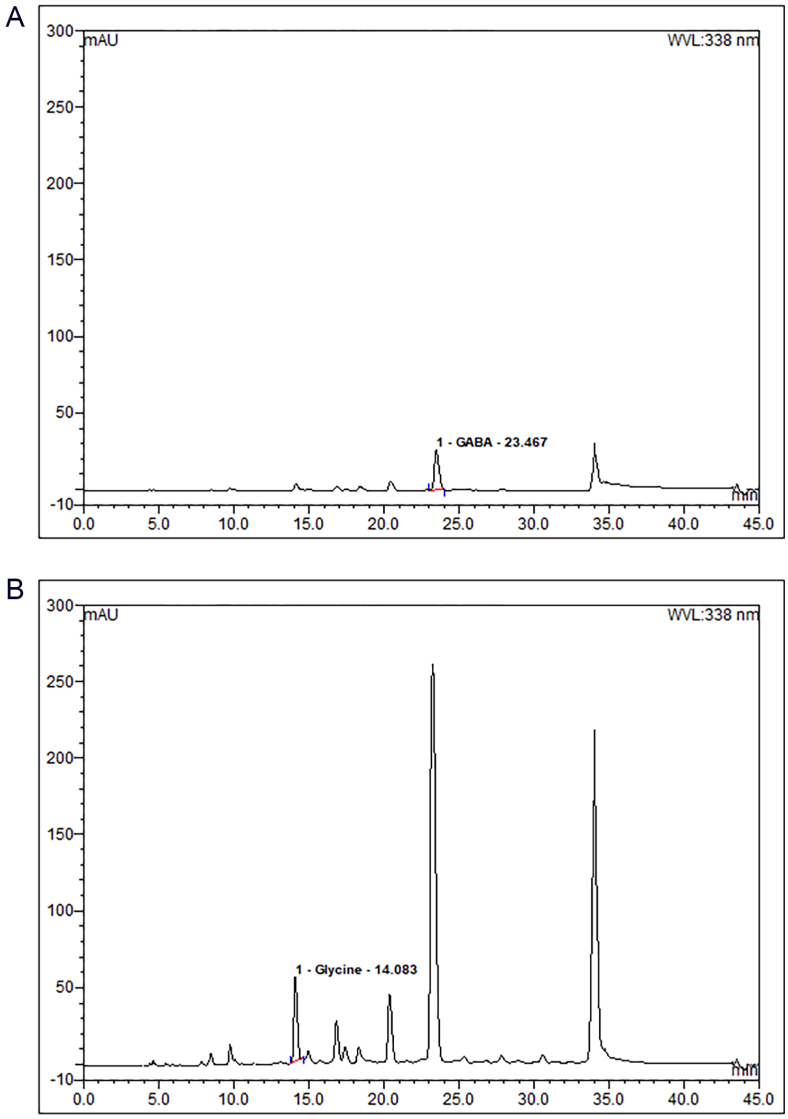

Figure S5. High-performance liquid chromatography analysis of GABA and Glycine in FC. FC, fermented fish collagen; GABA, gamma-aminobutyric acid; mAU, milli absorption unit; WVL, wavelength.

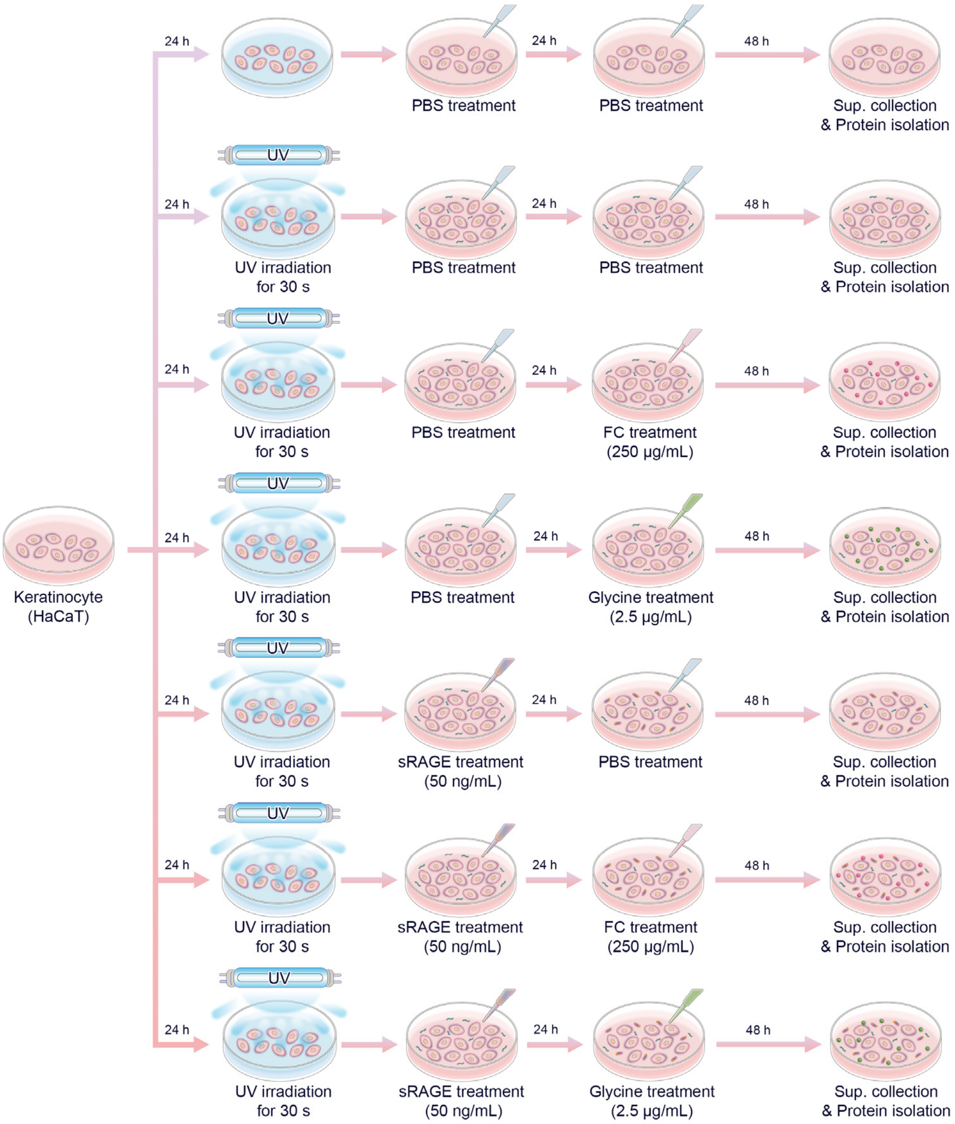

Figure S6. Schematic diagram showing sRAGE treatment followed by FC or glycine treatment of UV-exposed keratinocytes. FC, fermented fish collagen; h, hours; PBS, phosphate-buffered saline; s, seconds; sRAGE, soluble receptor for advanced glycation end product; Sup., supernatant; UV, ultra-violet.

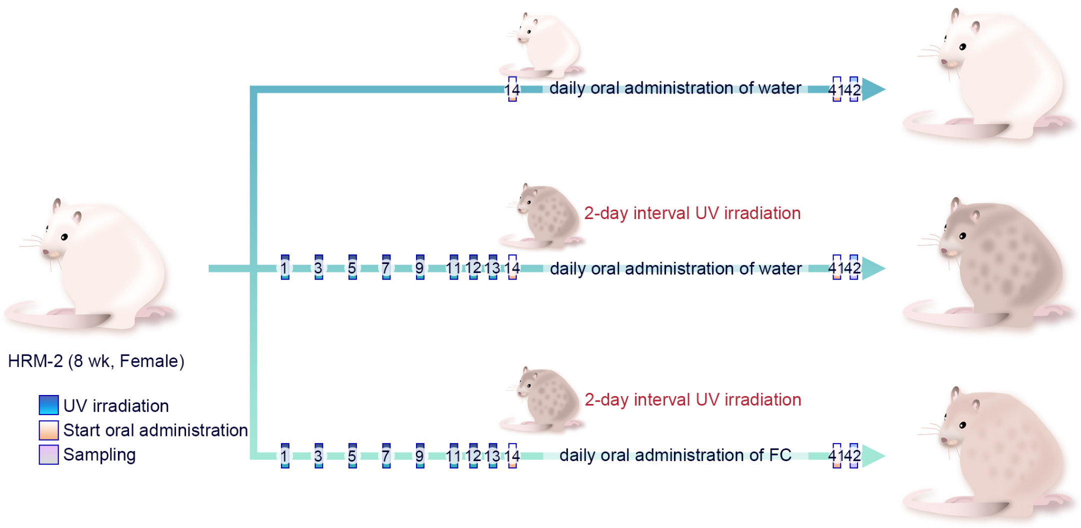

Figure S7. Schematic diagram showing the treatment of UV-exposed hairless mice skin with FC. FC, fermented fish collagen; UV, ultraviolet; wk, weeks.

Table S1. List of antibodies for enzyme-linked immunosorbent assay (ELISA), western blot (WB) and 3, 3'-diaminobenzidine staining (DAB).

Antibody	Company	Catalog No.	Dilution rate		
			ELISA	WB	DAB
TNF-α	Santa cruz	Sc-52746	1:100	-	-
AGE	Abcam	Ab23722	1:1,000	-	-
RAGE	Santa cruz	Sc-365154	1:100	-	-
NF-κB	Cell signaling	8242	-	-	1:200
MMP1	FINETEST	FNAB05223	-	1:1,000	-
MMP3	Abclonal	A1202	-	1:1,000	-
MMP9	Abclonal	A0289	-	1:1,000	-
Samd7	Santa cruz	Sc-365846	-	1:1,000	-
Smad2/3	Cell signaling	8685		1:1,000	
pSmad2/3	Cell signaling	8828	-	1:1,000	-
β-actin	Cell signaling	4967	-	1:1,000	-
Collagen 1	Santa cruz	Sc-293182	-	-	1:100
Collagen 3	Bioss	BS-0579R	-	-	1:100
